# Mirror Hand: An Uncommon Neglected Case Managed with Pollicisation

**Published:** 2017-05

**Authors:** Sunil Gaba, Naveen John, Sandeep Bhogesha, Onkar Singh, Guru Karna Vemula

**Affiliations:** Department of Plastic Surgery, PGIMER, Chandigarh, India

**Keywords:** Hand, Mirror, Neglected

**DEAR EDITOR**

Mirror hand or ulnar dimelia is a very rare congenital anomaly characterized by symmetric duplication of the upper limb in the midline. In most cases there is mirrored symmetry with a central digit and 3 digits on either side representing the middle ring and small fingers. The thumb is absent despite presence of seven digits. There is duplication of ulna and absence of radius. The preaxial ulna is often hypoplastic and supports the duplicated carpal elements.^1^ There are a few case reports of this rare anomaly. We report another case of mirror hand. It falls under I2bv according to IFSSH^2^ classification and type 1 in Al-Quattan classification.^3^

A 15 year old female patient presented with multiple fingers in her right hand with short forearm and radial deviation on flexion of the wrist. She was the first child of non consanguineous parents with normal antenatal history. None of her siblings had any congenital disorders. She had underwent corrective osteotomy with ring fixator for correcting the wrist flexion and radial deviation three years prior to reporting to us for management of polydactyly. She had seven digits in her right hand that were arranged symmetrically as mirror images on either side of a sagittal axis. There was no thumb present. The forearm was short with evidence of scars of previous surgery (Figure 1). 

**Fig. 1:**
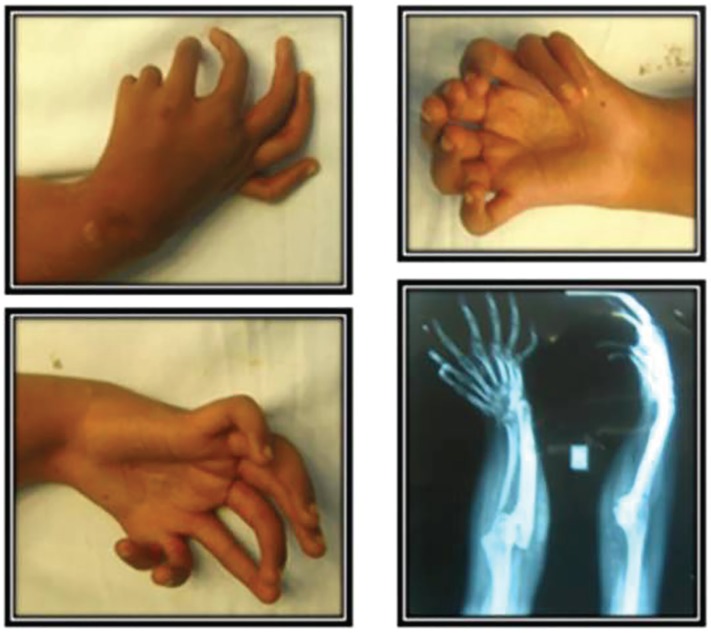
Pre operative clinical and radiological status

Movements at shoulder were grossly normal while that at the elbow and wrist were restricted in all range. On examination of function of hand the precise finger movements were absent and only crude grasp was evident. X-ray showed seven triphalangeal digits in right hand with corresponding metacarpals. There were two ulnae and both were shorter than the normal opposite limb. The peraxial ulna was hypoplastic with broad distal end that tapered proximally. The Radius was absent in the affected limb (Figure 1). In order to improve her hand function, it was decided to perform pollicisation. The third radial digit had more length and wider web space, and was therefore chosen for reconstruction of the thumb. The remaining two radial digits were amputated.

Technique of pollicisation: The lateral two digits were amputated; the skin incisions and fillet flaps were designed to maintain good vascularity and provide a wide first web space (Figure 2). The digital vessel of third ray was dissected and isolated. The communication between third and fourth ray at web were cut and ligated then shaft of third metacarpal removed sparing its head and base. The flexor and extensor tendon of third ray were shortened by plication. The intrinsic Muscles were atrophic for lateral two digits and hypoplastic for the third one.

**Fig. 2:**
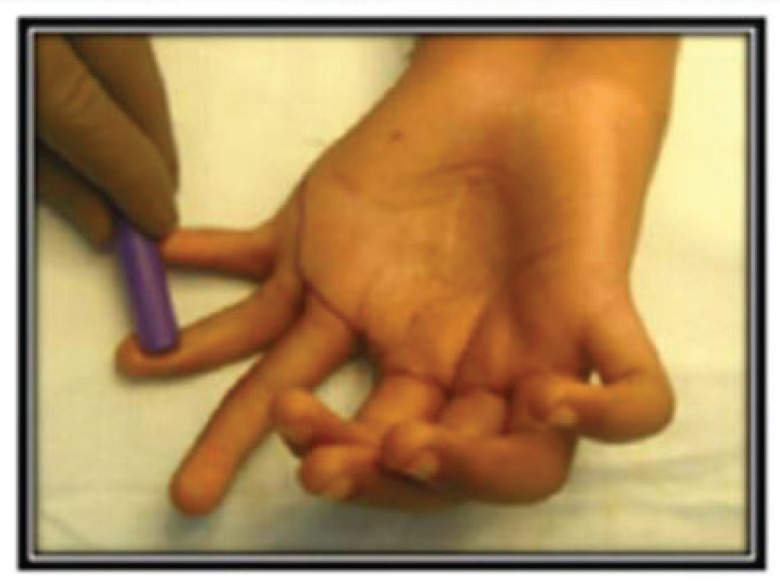
Intra operative markings

So, dorsal muscle with periosteal strip hitched to radial lateral band and palmar muscle mass attached to ulnar lateral bands. The third ray was abducted, pronated in desired position to serve as thumb function and fixed with K wires. The extra skin of fillet fl Figure 3 aps was used for deepening of first web space. On follow up satisfactory hand function was achieved () which is supported by DASH (Disability of the arm, shoulder and hand) score. Total DASH score is 12.93. 

**Fig. 3:**
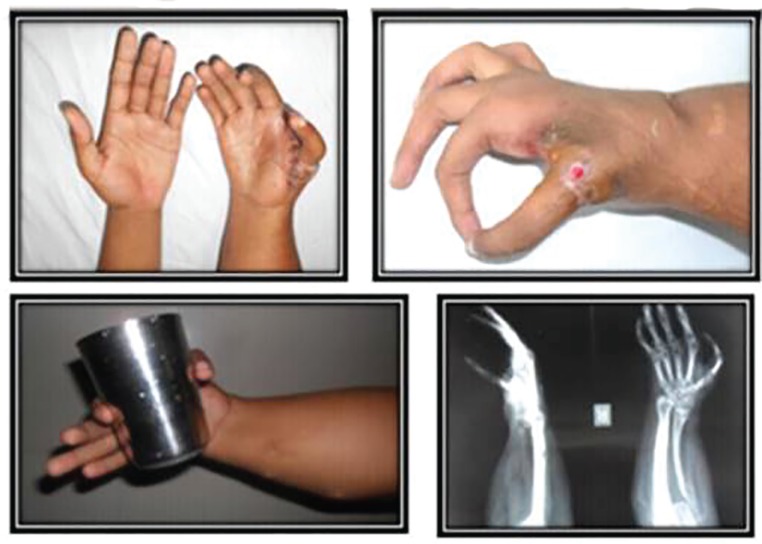
Post operative clinical, radiological and functional status

Mirror hand or ulnar dimelia is a rare congenital anomaly of the upper limb. Typically there are seven digits which are symmetrical along a sagittal axis with two ulnae and absent radius.^2^ The etiology of mirror hand has been attributed to the replication of zone of polarizing activity (ZPA) signaling center located in the posterior margin of the limb bud that controls the radio ulnar development through the signaling molecule, sonic hedgehog protein.^1^ Different anatomical presentation of mirror hand is seen with mirror hand.^4^^-^^7^ Al Quattan and Al-Thunayan have proposed a classification of mirror hand deformity based on the presence or absence of other congenital anomalies and the type of fore arm bones.^3^ Our case is type 1 according to their classification.

Aim in management of these patients is to achieve a functional and aesthetic upper limb and may involve a number of complex and multiple surgeries. The treatment is designed to reduce the number of digits to four and reconstruct a thumb from one of the most functional radial digit. The first web space can be augmented using the soft tissue from the amputated digits. 

## CONFLICT OF INTEREST

The authors declare no conflict of interest.
